# Frontal Alpha Asymmetry in Response to Stressor Moderates the Relation Between Parenting Hassles and Child Externalizing Problems

**DOI:** 10.3389/fnins.2022.917300

**Published:** 2022-07-05

**Authors:** Daniel J. Mulligan, Ava C. Palopoli, Marion I. van den Heuvel, Moriah E. Thomason, Christopher J. Trentacosta

**Affiliations:** ^1^Department of Psychology, Wayne State University, Detroit, MI, United States; ^2^Department of Cognitive Neuropsychology, Tilburg University, Tilburg, Netherlands; ^3^Department of Population Health, New York University Medical Center, New York, NY, United States; ^4^Department of Child and Adolescent Psychiatry, New York University Medical Center, New York, NY, United States; ^5^Neuroscience Institute, NYU Langone Health, New York, NY, United States

**Keywords:** frontal alpha asymmetry (FAA), EEG, externalizing problem behavior, parenting daily hassles, child, urban, stress, family environment

## Abstract

Inequitable urban environments are associated with toxic stress and altered neural social stress processing that threatens the development of self-regulation. Some children in these environments struggle with early onset externalizing problems that are associated with a variety of negative long-term outcomes. While previous research has linked parenting daily hassles to child externalizing problems, the role of frontal alpha asymmetry (FAA) as a potential modifier of this relationship has scarcely been explored. The present study examined mother-child dyads, most of whom were living in low socioeconomic status households in an urban environment and self-identified as members of racial minority groups. Analyses focused on frustration task electroencephalography (EEG) data from 67 children (mean age = 59.0 months, SD = 2.6). Mothers reported the frequency of their daily parenting hassles and their child’s externalizing problems. Frustration task FAA moderated the relationship between parenting daily hassles and child externalizing problems, but resting FAA did not. More specifically, children with left frontal asymmetry had more externalizing problems as their mothers perceived more hassles in their parenting role, but parenting hassles and externalizing problems were not associated among children with right frontal asymmetry. These findings lend support to the motivational direction hypothesis and capability model of FAA. More generally, this study reveals how individual differences in lateralization of cortical activity in response to a stressor may confer differential susceptibility to child behavioral problems with approach motivation (i.e., left frontal asymmetry) predicting externalizing problems under conditions of parental stress.

## Introduction

By 2050, 69% of people will live in urban areas; yet these environments are marked by social inequality and (re)produce conditions of threat, deprivation, and toxic stress associated with increased incidence of anxiety, mood disorders, and altered neural social stress processing (Kiser, 2007; Lederbogen et al., 2011). A 2014 Substance Abuse and Mental Health Services Administration report documented a recent decline in America’s public health due to the preponderance of behavioral health conditions developmentally rooted in toxic stress and trauma (Shern et al., 2016), marking a new era in public health. Because complex urban ecologies stratify resources and stressors along economic and racialized lines, they disproportionately subject children from poor and/or racial minority families to toxic stress, resulting in health disparities (Szanton et al., 2005; Allen et al., 2014; Shonkoff et al., 2021). Understanding and mitigating the toxic effects of urban ecological stress is therefore not only a matter of great concern for science, but also public health, social justice, and public policy.

Self-regulation is arguably the most fundamental psychosocial capacity youth must develop. Consequently, when toxic stress disrupts its development, the basis for lifelong biopsychological, academic, and occupational functioning is undermined (Shonkoff et al., 2012; Murray et al., 2015). In children, for example, emotion regulation (a facet of self-regulation) predicts social competence, school performance, inhibition of aggression, empathy, and prosocial behavior (Denham et al., 2003; Izard et al., 2008; Trentacosta and Shaw, 2009). Conversely, emotion dysregulation disrupts socially appropriate goal-directed behavior and has been well-established as a risk factor for and symptom of many forms of psychopathology (Keenan, 2000; Beauchaine, 2001; Cole and Hall, 2008; Aldao et al., 2010; Nolen-Hoeksema, 2012). Externalizing problems are one of the more deleterious consequences of the disruption of self-regulation development (Farahmand et al., 2011; Lansford et al., 2011; Pearl et al., 2012; Flouri and Sarmadi, 2016). Early-onset externalizing behaviors convey risk for an array of developmental problems such as social rejection, early dropout, and developmental cascades toward diagnoses of disruptive behavior disorders, academic dysfunction, substance abuse, and criminality (Masten et al., 2005; Petersen et al., 2015; Paz et al., 2021). Thus, understanding externalizing problems in young children is vital.

The Bioecological Theory of Development (Bronfenbrenner, 2005) can be used to better understand how early childhood externalizing problems develop in interaction with environmental stress, parenting, and neural processes. The Bioecological Theory explains child developmental outcomes as the product of the simultaneous and synergistic effects of four primary factors: proximal processes, person characteristics, contexts, and time (the PPCT model). Bronfenbrenner conceptualized a dysfunctional outcome as “the recurrent manifestation of difficulties in maintaining control and integration of behavior across situations and different domains of development” (Bronfenbrenner and Evans, 2000, p. 118). In the case of childhood externalizing problems, the lack of control takes the form of hyperactive, inattentive, rule-breaking, and aggressive behaviors, which tend to cluster together due to putatively shared proximal and distal causes. The empirical literature suggests these proximal and distal causes include child factors such as temperament and executive functioning (Dodge et al., 2007), parenting factors such as insensitivity (Halligan et al., 2013) and harsh control (Pinquart, 2017), and environmental factors such as poverty, unstructured settings, and stressful life events (Hicks et al., 2009). The PPCT model can be used to sort this data into a theoretical framework that generates testable hypotheses.

According to the Bioecological Theory, proximal processes drive developmental change through repeated reciprocal interactions between the developing child and the people, objects, and symbols in their environment, leading to the actualization of genetic potential into progressively complexified phenotypes. These proximal processes are the activities, social roles, and interpersonal relations taking place in the child’s microsystems, the physical contexts wherein these face-to-face interactions regularly occur (e.g., the home, classroom, or playground). The system of microsystems (i.e., mesosystem) is embedded within the exosystem, the ecological settings in which the child does not participate but is nonetheless indirectly causally connected (e.g., parental workplace affecting familial relationships and vice versa). Finally, the exosystem is nested within the macrosystem, the economic, cultural, educational, and legal systems (among others) that are structured by ideologies and together regulate the more proximal systems, conferring similar experiences to children of the same class, race/ethnicity, religion, etc. One example of an empirically supported framework that explains child developmental outcomes in terms of overlapping processes in the exosystem and microsystem is the Family Stress Model (Conger et al., 2000; Masarik and Conger, 2017). It posits that child behavior problems arise from disrupted parenting and interparental conflict (i.e., proximal processes in the microsystem), which arise from parental distress caused by economic pressures (i.e., exosystem processes), which are caused by economic and social policy (i.e., macrosystem processes), all of which are modulated by risk and protective factors. Within this family stress literature, many studies have reported a connection between daily parenting hassles and child externalizing problems (Crnic and Greenberg, 1990; Creasey and Reese, 1996; Gülseven et al., 2018). Because these effect sizes tend to fall in the medium range, other aspects of the PPCT model can explain how and why frequent parenting hassles cooccur with frequent child externalizing problems for some families but not others. In the context of toxic urban stress and given the evidence for differential susceptibility (Belsky and Pluess, 2009), person characteristics of the child likely play a pronounced role.

Daily parenting hassles are minor but frequent stressors faced when completing parenting tasks and managing challenging child behaviors. In contrast to stressful life events such as a job loss or death in the family, daily parenting hassles are relatively minor stressors such as being whined at, cleaning up messes, maintaining child schedules, and keeping an eye on the children that can nonetheless accumulate to present a major ongoing challenge to parents. Parenting hassles predict behavioral and psychological problems in young children, sometimes more strongly than stressful life events (Kanner et al., 1981; Crnic and Greenberg, 1990; Creasey and Reese, 1996; Coplan et al., 2003; Crnic et al., 2005; Gülseven et al., 2018; Taylor, 2019). More specifically, they have predicted externalizing problems in diverse samples of children ranging from 2 to 12 years old (Kliewer and Kung, 1998; Coplan et al., 2003; Crnic et al., 2005; Yaman et al., 2010; Stone et al., 2016; Walerius et al., 2016). These hassles are thought to disrupt effective parenting, dyadic co-regulation of emotion, and productive activity by causing parents to become more impatient, irritable, and self-focused. Because these dyadic interactions are reciprocal, the effects are bidirectional (Pearl et al., 2012), with child externalizing behaviors and “difficult” temperament also increasing parenting behaviors and perceived stress (Chang et al., 2004; Walerius et al., 2016), resulting in feedback loops. Because the specific disrupted parenting behaviors that mediate the relation between hassles and child behavior problems likely depend upon unique characteristics of a given sample, studying daily parenting hassles as a main predictor is beneficial. Consistent with the Bioecological Theory, studies have found that person and contextual characteristics modify the relationship between parenting hassles and child externalizing problems. For example, in a sample of families living in an under-resourced urban area, high family cohesion, routines, and adaptability attenuated the relationship between parenting daily hassles and externalizing problems (Kliewer and Kung, 1998). In line with the PPCT model, person characteristics that confer direction and power to proximal processes may modify the association between parenting hassles and externalizing problems. These person characteristics include dispositions, which are thought to catalyze and sustain proximal processes, and demand characteristics, which attract or repel others in the child’s microsystems to aid or hinder proximal processes. Child temperament is one especially relevant disposition and demand characteristic. In a study of preschool children, inattentive and shy temperamental dispositions predicted externalizing problems, and higher inattentiveness strengthened the relationship between parenting hassles and externalizing problems (Coplan et al., 2003). Frontal alpha asymmetry (FAA), an EEG measure of hemispheric asymmetry in the activity of the prefrontal cortex under resting or emotionally challenging conditions, is a potential demand characteristic that is also a marker of children’s temperamental dispositions (Howarth et al., 2016; Anaya et al., 2021; Vincent et al., 2021). However, FAA has not yet been studied as a factor that might moderate the association between parenting daily hassles and children’s externalizing problems.

Pioneering research on FAA began over 40 years ago (Davidson et al., 1979), resulting in myriad published studies that have investigated a variety of constructs in models of emotion regulation, motivation, temperament/personality, and psychopathology (see Coan and Allen, 2004; Harmon-Jones et al., 2010; Saby and Marshall, 2012; Peltola et al., 2014; Allen and Reznik, 2015; Kelley et al., 2017; Reznik and Allen, 2018). FAA is typically calculated as the difference in the natural logs of the alpha power band between homologous right and left frontal electrodes, most commonly the midfrontal (F3-F4) or lateral frontal (F7-F8) (Reznik and Allen, 2018). Because alpha oscillations (typically measured at ∼8–10 Hz in children and 6–9 Hz in infants) are thought to inhibit cortical network activity (Pfurtscheller et al., 1996; Coan and Allen, 2004; Mathewson et al., 2011), researchers conventionally assume that FAA indirectly indexes asymmetry in cortical activity, such that more positive FAA scores (ln[right] − ln[left] > 0) indicate relatively more left frontal activity and vice versa. Perhaps confusingly named, FAA likely measures asymmetry in the prefrontal cortex (PFC), not (only) the frontal cortex, and predominately the dorsolateral prefrontal cortex (dlPFC). Though many studies omit this fact, it was explained by Davidson (2004) and later elaborated by Grimshaw and Carmel (2014), who pointed out that alpha oscillations are especially linked to inhibition of the dlPFC (Laufs et al., 2003; Pizzagalli et al., 2005; Mantini et al., 2007; Koslov et al., 2011). For the sake of brevity and clarity, we will henceforth discuss the direction of asymmetry in terms of cortical activity/activation (rather than alpha power, which is inversely related), such that *left asymmetry* refers to relatively more left than right dlPFC activity (as indexed by alpha power; i.e., right FAA; FAA > 0) and *right asymmetry* refers to relatively more right than left dlPFC activity (i.e., left FAA; FAA < 0).

A large body of research supports the motivational direction model of FAA in which one’s FAA score reflects a relatively stable disposition toward approach- or withdrawal-oriented affect and behavior (Harmon-Jones and Gable, 2018). Right asymmetry corresponds to withdrawal-oriented motivational states [e.g., negative affect such sadness, fear, and disgust (Davidson, 2003)] and traits [e.g., child temperament factors such as behavioral inhibition (Goldstein et al., 2019), social avoidance, and non-positive shyness (Poole and Schmidt, 2020)]. This withdrawal tendency can become extreme and maladaptive in depressive disorders, for which right asymmetry is a neurophysiological endophenotype (Stewart et al., 2010; Allen and Reznik, 2015). Conversely, left asymmetry corresponds to approach-oriented motivational states [e.g., anger (Harmon-Jones and Allen, 1998; Harmon-Jones, 2007) and positive affect (Rutherford and Lindell, 2011)] and traits [e.g., higher self-reported behavioral activation system scores, more focus on promotion than prevention, higher reward sensitivity (Harmon-Jones and Gable, 2018), positive urgency (Gable et al., 2015), and activity level (Howarth et al., 2016)]. This disposition toward approach motivation has more often been studied as an adaptive factor, protecting against risk for depression, increasing in response to treatments for depression, and marking adaptive emotion regulation (Reznik and Allen, 2018).

Comparatively less attention has been paid to left asymmetry’s relation to forms of dysregulated and maladaptive approach motivation. While left asymmetry is often associated with approach behaviors and positive emotions, it can also become maladaptive and link to inhibition issues. For instance, left asymmetry has been associated with ADHD (Hale et al., 2009; Keune et al., 2015; Ellis et al., 2017), violent aggression (Peterson et al., 2008; Keune et al., 2012), hostility in people with borderline personality disorder (Beeney et al., 2014), and hypomanic/manic symptoms in those with bipolar disorder (Nusslock et al., 2015). In all cases, left asymmetry is likely marking maladaptive approach motivation, be it impulsivity, hyperactivity, socially inappropriate anger and aggression, or distraction by rewarding/appetitive stimuli. Meta-analyses show no consistent predictive relationship between FAA and externalizing problems in children, at least when FAA is measured in resting state (Peltola et al., 2014; Perizzolo et al., 2017); however, consistent with stress-diathesis and differential susceptibility models, multiple studies have found FAA interacts with other risk factors to predict psychopathology. For example, a temperament measure of impulsivity-anger predicted more child externalizing problems only for children with more left asymmetry (Liu et al., 2021). For high-risk children, left asymmetry attenuated the association between stress and internalizing problems (Lopez-Duran et al., 2012). Gatzke-Kopp et al. (2014) found left asymmetry predicted externalizing problems only in the presence of heightened physiological reactivity, which supported an efferent filter model of EEG asymmetry. According to this model, left asymmetry simply biases behavioral responses to emotional reactions to emotionally challenging stimuli, making it only confer risk to externalizing problems in interaction with contextual risk (e.g., low SES and low verbal ability). Applying the efferent filter model of FAA to the PPCT model, FAA may serve as a dispositional bias that interacts with contextual risk factors in the microsystem to produce problem behaviors under specific conditions.

Although it has not been studied, it stands to reason that FAA in children may interact with daily parenting hassles to predict externalizing problems, especially in the context of cumulative environmental risk. When a child has a withdrawal-oriented disposition and their parents more frequently subjectively perceive their parenting role as a hassle, they may experience heightened fear, sadness, or anxiety and consequently avoid conflict with their parents. On the other hand, a child with an approach-oriented disposition may respond to parental stress by becoming more labile, impulsive, and/or inattentive, which may intensify their parents’ stress. Thus, left asymmetry may simultaneously function as a demand characteristic, producing difficult behaviors that evoke impulsive, ineffective parenting tactics and deter more empathetic, sensitive responding.

Research suggests left asymmetry’s associations with maladaptive approach-oriented behaviors are more likely to manifest when FAA is measured during an emotionally challenging task rather than in an eyes-closed resting condition. FAA was originally conceptualized as *affective style*, a measurement of individual differences in resting state asymmetry representing a “broad array of processes that either singly or in combination modulate an individual’s response to emotional challenges, dispositional mood, and affect-relevant cognitive processes” (Davidson, 2000). Then, citing the problems of uncontrolled situational factors, idiosyncratic participant mental behavior during resting conditions, and FAA’s inconsistent correlations with personality traits, Coan et al. (2006) proposed an alternative *capability model* of FAA which “posits that meaningful individual differences in frontal EEG asymmetry exist, but that those individual differences are best thought of as interactions between the emotional demands of specific situations and the emotion-regulatory *abilities* individuals bring to those situations” (p. 198). Many subsequent studies have found incremental predictive validity for the capability model (Crost et al., 2008; Goodman et al., 2013; Stewart et al., 2014; Harmon-Jones and Gable, 2018; Reznik and Allen, 2018). It follows that if externalizing problems are the behavior of interest, it makes sense to measure FAA during an emotionally challenging task designed to elicit a specific dlPFC response relevant to the proximal and/or distal causes of externalizing problems. If designed successfully, it may then demonstrate superior predictive validity compared to FAA measured in resting state.

The current study sought to examine how parenting and child cortical processes interact to give rise to childhood externalizing problems. To address this aim, we measured child FAA during two relatively novel conditions: a resting-state condition and an emotionally challenging condition. Our first hypothesis was that child FAA in the emotionally challenging condition would moderate the relationship between parenting daily hassles and child externalizing problems, such that the association would be obtained for children with left asymmetry but not for those with right asymmetry. Second, we hypothesized that this moderation effect would not be replicated with FAA measured during the resting state condition.

## Materials and Methods

### Procedure

The current study is part of an ongoing, larger longitudinal study that has followed mothers and their children from 20 to 40 weeks gestation. Participants were recruited at a hospital during routine prenatal care appointments in Detroit, MI, United States. Prenatal care physicians introduced expecting mothers to a study involving fetal MRI during pregnancy, and mother-child assessments were conducted postnatally, including longitudinal visits during infancy, toddlerhood, and at age 5 years. Eligible pregnant women for the larger study were age 18–40 years old, had singleton pregnancies, spoke English as a first language, and showed no MRI contraindications, which was a focus of the larger project. The current study focused on data obtained at the age five visit. During the age five visit, mothers completed questionnaires that included demographic information, their feelings on parenting, and ratings of children’s behavior problems. EEG data were also collected from the children at the age five visit. The study was approved by the Institutional Review Board of Wayne State University. All mothers signed informed consent documents prior to participation.

### Participants

Participants in this study were drawn from an ongoing, longitudinal study of child development based in Detroit, Michigan. All children who completed the age five study visit at the time of this analysis were eligible. Of those eligible (*n* = 110), 15 were removed due to technical problems during collection, 19 opted out or were non-compliant, and 6 had missing EEG data for undocumented reasons. An additional three EEG datasets were excluded due to presence of too few artifact-free bins. This left a total sample of 67 children with quality assured data for the frustration task and 68 children with quality assured data for the resting task.

Mothers were 30.3 years old on average (*SD* = 4.7) at the time of the age five visit. Mean gestational age at birth was 38.8 weeks (*SD* = 1.6), and mean age for the child at the age five visit was 59 months (*SD* = 2.6). Children were 43.3% female and 56.7% male. Self-report data of maternal racial identity showed the sample was predominantly African American (80.6% African American, 11.9% White, 4.5% Multiracial, 1.5% Latina, and 1.5% Asian American). Additional maternal self-report data further illustrated that the current sample was of a low socioeconomic status; 54.5% of mothers reported gross annual household income of $20k or less, 67.2% reported receiving public assistance, 14.1% had no GED or high school diploma, and 84.6% lived in census tracts ranked in the bottom quintile of the nationally normed Childhood Opportunity Index (Acevedo-Garcia et al., 2020).

### Measures: Maternal Self-Report Questionnaires

#### Parenting Daily Hassles

The PDH (Crnic and Greenberg, 1990) is a 20-item survey of everyday stressors due to parenting tasks or difficult child behaviors, such as cleaning up messes or children demanding entertainment. Mothers rated each item for both frequency of event occurrence (*rarely, sometimes, a lot, constantly*) and intensity of hassle (using a 5-point Likert scale). In the original PDH study, factor analysis led to the creation of two subscales: Parenting Tasks (factor one) and Challenging Behavior (factors two and three combined) (Crnic and Greenberg, 1990). The Parenting Tasks subscale is comprised of eight items about typical tasks or duties enacted by parents (e.g., “Having to change your plans because of unexpected child needs”). The seven-item Challenging Behavior subscale asks about child behaviors that parents commonly find difficult to manage (e.g., “The kids won’t listen or do what they are asked without being nagged”). The five remaining items comprising the PDH are not part of either subscale. Summary scores can be created to determine the frequency and intensity of Parenting Tasks, Challenging Behaviors, and/or the total PDH scale. Previous research has shown adequate reliability and validity for the PDH to measure parenting hassles (Crnic and Greenberg, 1990; van IJzendoorn et al., 2008; Smith, 2011; Finegood et al., 2017). Eight participants in the current sample were missing frequency data, and 23 were missing intensity data. Because more frequency than intensity data were available in the current sample and previous studies have found predictive validity for total frequency of parenting daily hassles (Stone et al., 2016), the PDH Total Frequency scale was used for the primary analysis (i.e., the sum of frequency scores for all 20 items). For sensitivity analyses described below, frequency of Parenting Tasks was also calculated and analyzed.

#### Child Behavioral Checklist 1.5–5

The CBCL Achenbach and Rescorla (2001) is a widely used, well-validated, 99-item questionnaire in which mothers reported the perceived frequency of their child’s behavioral problems (0 = *not true*, 1 = *somewhat or sometimes true*, 2 = *very true or often true*). The CBCL includes two broad-band scale scores, the 36-item Internalizing Problems scale (e.g., “unhappy, sad, or depressed”) and the 24-item Externalizing Problems scale (e.g., “hits others”), which are reflective of the sum of the items for each scale. The two scales show adequate to high internal consistency, adequate test-retest reliability, and adequate evidence of construct validity (Achenbach and Rescorla, 2000; Rescorla, 2005; Ivanova et al., 2010; Carneiro et al., 2016).

#### Covariates

Variables used as covariates in the current study were child sex, gestational age at birth, gross annual household income, maternal age at the age five visit, and maternal symptoms of anxiety and depression. Covariates were selected based on their links to externalizing problems in children: theoretically, empirically in the literature, and in terms of the correlation coefficient in this sample. Child sex and gestational age at birth were obtained from birth records. Mothers reported their household income during the age five visit. Maternal age at the date of the age five visit was calculated from previously reported dates of birth. Maternal anxiety and depressive symptoms were measured with the State Trait Anxiety Inventory—Trait Scale (STAI-T; Spielberger et al., 1983), which was administered to mothers at the age five visit. Derived from the 40-item State-Trait Anxiety Inventory (STAI), the STAI-T is a 20-item standardized measure of trait anxiety, or anxiety as a personality feature (Bieling et al., 1998; Dubber et al., 2015), although it is also recognized as a measure of symptoms of anxiety and depression (Bados et al., 2010). Items on the STAI-T are designed to evaluate the frequency of feelings “in general” on a 4-point intensity scale ranging from 1 = *almost never* to 4 = *almost always*. The STAI-T has demonstrated strong internal consistency (α = [0.86, 0.92]), adequate construct validity (Spielberger et al., 1983; Barnes et al., 2002), and test-retest reliability (*r* = 0.84 for men and *r* = 0.76 for women).

### Measures: Electroencephalography Tasks

#### Emotionally Challenging Condition: Incredible Cake Kids Electroencephalography Task

The Incredible Cake Kids task was a computer game played during EEG data collection (Grabell et al., 2019). Children were told that just like some kids are good at sports, some kids are good at baking cakes; a video subsequently instructed them that they would work in a cake shop and choose the best cake of three options for each customer. The cartoon customers reacted by either liking the cake, smiling, and exclaiming enthusiastically (e.g., “Yummy!” or “Oh, yea!”), or disliking the cake, frowning, and exclaiming in disgust or disappointment (e.g., “Yuck!” or “Oh, no…”). [For screenshots of the task, see Grabell et al. (2019)]. These responses were unrelated to the selected cake and randomized, so children could neither control nor predict the feedback. The same randomized order was presented to all children. The negative evaluation was intended to be a developmentally appropriate, mild social evaluative stressor. Following a practice round of three trials, children played the game, experiencing a ratio of 12 positive feedback trials to 18 negative feedback trials. After the final practice trial (positive feedback), feedback trial 10 (negative feedback), trial 20 (negative feedback), and trial 30 (positive feedback), children were asked how they felt on a 1 (“very sad”) to 7 (“very happy”) emoji scale. The seven emojis were presented in order from very sad to very happy on the computer screen, and children were instructed to point to the emoji showing how they presently felt. The practice round presented negative feedback once (trial 2), whereas in the game, trials 1–10 consisted of 60% negative feedback, trials 11–20 were 70% negative, and trials 21–30 were 50% negative.

#### Resting-State Condition: Pokémon-Themed Resting, Eyes-Closed Electroencephalography Task

We developed a novel EEG protocol designed to produce quiescent behavior in children so that EEG data could be acquired in a quiet, still, eyes-closed state for eight consecutive 25-s epochs. Children were told a Pokémon would hatch on the computer screen if they put their chin on a toy egg incubator and kept very still with their eyes closed until they heard a bell sound effect. This behavior was first modeled by an experimenter with the Pokémon animation playing on the screen. The animation consisted of an egg sitting still for 25 s until a bell sounded followed by a hatching sound and an animation of a new Pokémon emerging from the egg and proclaiming its name. The child then followed this protocol for eight consecutive 25-s trials, each resulting in a new Pokémon hatching. When the bell sounded, the experimenter instructed the child to open their eyes and watch the animation. The child then received a sticker corresponding to the hatched Pokémon and placed it on their visit checklist. This procedure was repeated as an attempt to collect eight 25-s trials of restful, quiet, eyes-closed EEG data. During this task, children were videotaped along with a video of the computer screen so that child behavioral compliance could subsequently be assessed.

### Electroencephalography Data Collection and Processing

Children wore an electrode cap configured to the 10–20 system and the standard BioSemi reference scheme (CMS-DRL) with electrodes on the two mastoids. A 34-electrode system with BioSemi ActiveTwo amplifiers recorded the EEG signal with a 2048 Hz sampling rate. To check for protocol compliance, research assistants coded videos of children completing the EEG tasks. Trials of the Incredible Cake Kids frustration task were coded as positive feedback, negative feedback, missing, or not looking, and discarded when the participant did not respond (i.e., missing) or was not looking during feedback stimulus presentation. For the Pokémon condition, research assistants coded 25-s resting state segments as mistrials whenever the child opened their eyes, squeezed their eyes together, opened their mouth wide, raised their eyebrows high, got out of their seat, or when their eyes were not visible in the frame. These mistrials were discarded.

After recording, EEG data were preprocessed with BrainVision Analyzer software and run through a 60 Hz notch filter and a 0.1–30 Hz band-pass filter. All electrode signals were screened for data quality. Signals were referenced to the mastoid electrodes. Interpolation was used in some cases to replace electrode signals with extremely large amplitudes and low frequency waves, suggesting a misconfigured electrode. In one case, it was necessary due to distortions to use only one mastoid electrode for reference. The F3/F4 and F7/F8 electrode pairs were inspected for FAA calculations. The F7/F8 electrodes were ultimately not used to calculate FAA because the electrodes contained a relatively higher number of artifacts, likely due to forehead and eyelid muscle proximity.

For the frustration task, the F3 and F4 electrode data were segmented into 2-s post stimulus bins and only bins for negative feedback trials were used. Thus, the maximum number of 2-s bins per participant was 18. For the Pokémon condition, the 25-s segments were further segmented into 2-s bins using a hamming window with 50% overlap. Blink correction was not performed. The bins with a voltage change greater than 75 μV/ms or a greater range than 200 μV were removed from analysis using automatic artifact rejection. Three cases with less than 10 artifact-free bins for the frustration task condition were excluded from the study. There was an average of 14.9 artifact-free bins used in the frustration task condition and an average of 119.0 in the resting condition. To extract alpha power, remaining bins underwent Fast Fourier Transformation. Alpha range was defined as 6–9 Hz in line with past EEG research with low socioeconomic status and African American samples (Marshall et al., 2002; Ehlers and Phillips, 2003; Otero et al., 2003). Average alpha power for F3 and F4 was calculated for each subject for all available bins. FAA was computed as the difference between the log-transformed alpha powers of the right and left frontal electrodes (ln[F4]–ln[F3]), per condition.

### Statistical Approach

We used SPSS 28 to perform all analyses. After checking descriptive statistics, univariate outliers, and missing data, we used multiple imputation to deal with missingness. We inspected data for violations of the assumptions of the general linear model (e.g., checking histograms, skewness, kurtosis, mahalanobis distances, P-P plots, scatterplots of residuals by predicted scores, etc.). Sequential multiple regression was used to test the significance of parenting daily hassles, frustration task FAA, and their interaction as predictors of child Externalizing Problems. The significant interaction was probed with a follow-up simple slopes analysis and tests distinguishing the type of interaction effect (i.e., differential susceptibility vs. diathesis-stress). These regressions were rerun with covariates added. We then used sequential multiple regression again to test the significance of parenting daily hassles, *resting* FAA, and their interaction in an analysis that included covariates. Finally, a sensitivity analysis was run with the PDH Parenting Tasks subscale to verify that the relationship between parenting daily hassles and externalizing problems was due to more than conceptual redundancy of the constructs.

## Results

### Data Cleaning, Missing Data, and Multiple Imputation

Primary analyses included all cases (*N* = 67) with usable EEG data from the Incredible Cake Kids frustration task. PDH Total Frequency was positively skewed (skew = 1.07), but transformation was not considered necessary due to the slight elevation above the common threshold of 1. This decision was reinforced when regressions were later run, and residuals were normally distributed. There were no univariate outliers, and no variables were transformed for the analyses. Because more than a third of participants were missing EEG data and FAA was of primary empirical interest, we chose not to impute these values so that inferences about EEG data would therefore only be drawn from empirically observed cases. Missingness from other variables ranged from 0 to 15.5% of cases. Multiple imputation created 10 imputed datasets with complete questionnaire data for all 110 participants who completed the age five visit using the automatically determined method of imputation, a maximum of 100 case draws, and a maximum of five parameter draws. Variables with missing data were constrained to only accept values within the scale range. This imputation allowed analyses of frustration task FAA (*N* = 67) and resting FAA (*N* = 68) to have complete data. Unless otherwise stated, the effects reported were pooled from the 10 imputed datasets. Descriptive statistics can be seen in Table 1.

**TABLE 1:** Descriptive statistics.

					Frustration task EEG			Resting EEG		
	CBCL EP	PDH	PDH–CB	PDH–PT	FAA	F3	F4	FAA	F3	F4
Mean	9.43	35.19	12.33	12.73	0.02	4.60	4.62	0.11	5.05	5.59
Median	8.00	34.00	12.00	11.00	0.04	4.22	4.39	0.12	4.62	5.13
SD	7.55	8.56	4.76	4.61	0.20	2.13	1.77	0.14	2.05	2.21
Skewness	1.05	1.07	0.61	0.93	–0.46	2.02	1.17	–0.28	0.88	1.03
Kurtosis	0.88	1.79	–0.56	0.08	0.05	5.64	1.44	0.93	–0.19	0.28
Minimum	0.00	20.00	7.00	8.00	–0.49	1.72	1.72	–0.28	2.27	2.68
Maximum	33.00	61.00	23.00	24.00	0.40	13.66	10.35	0.48	9.90	11.13

*CBCL–EP, Child Behavior Checklist externalizing problems scale; PDH, Parenting Daily Hassles total frequency scale; PDH–CB, Parenting Daily Hassles challenging behaviors frequency subscale; PDH–PT, Parenting Daily Hassles parenting tasks frequency subscale; FAA, frontal alpha asymmetry; F3, electrode F3 alpha power; F4, electrode F4 alpha power.*

### Frustration Task Electroencephalography Condition

#### Child-Reported Emotional Valence

Average child-reported emotional valence (on 1–7 emoji scale) was 6.32 (*SD* = 1.68) after the final practice trial (positive feedback), 4.76 (*SD* = 2.71) after trial 10 (negative feedback), 5.15 (*SD* = 2.30) after trial 20 (negative feedback), and 5.48 (*SD* = 2.08) after trial 30 (positive feedback). Emotional valence decreased significantly from the final practice trial to trial 10, *t*(1, 59) = −4.70, *p* < 0.001. From trial 10 (negative feedback) to trial 20 (negative feedback), ratings did not change significantly (two sided *p*), *t*(1, 61) = 1.15, *p* = 0.25. Ratings also did not change significantly from trial 20 (negative feedback) to trial 30 (positive feedback), *t*(1, 61) = 0.89, *p* = 0.38. Emotional valence ratings were significantly lower at the end of the game (trial 30) than immediately prior to the game (final practice trial), *t*(1, 59) = −2.69, *p* = 0.005. The cumulative mean of the three in-game ratings was 5.13 (*SD* = 1.69) with a mode of 7. Average emotional valence after the two positive trials was 5.88 (*SD* = 1.44) whereas average emotional valence after the two negative trials was 4.89 (*SD* = 2.15). Average emotional valence after the two negative trials was significantly lower than average emotional valence after the two positive trials, *t*(1, 59) = −3.46, *p* < 0.001.

#### Correlations and Main Effects

Pearson’s product-moment correlation coefficients were calculated, revealing a significant linear relationship between the PDH Total Frequency scale and child Externalizing Problems scale (*r* = 0.33, *p* = 0.009), but not between frustration task FAA and Externalizing Problems (*r* = 0.03, *p* = 0.81) or FAA and PDH Total Frequency (*r* = 0.15, *p* = 0.29). FAA values measured in the frustration task and during the Pokémon-themed resting, eyes-closed condition were moderately correlated, *r* = 0.31, *p* = 0.02. Frustration Task FAA and average child-reported emotional valence during the task were not correlated, *r* = 0.03, *p* = 0.80.

Sequential multiple regression was used to first test the significance of main effects and then the interaction effect. The main effect model was significant, *F*_(2_,_64)_ = 3.99, *p* = 0.02, accounting for 11.1% of the variance in Externalizing Problems (*R* = 0.33, *R*^2^ = 0.11). PDH Total Frequency was a significant predictor, *b* = 0.29, *se* = 0.11, *t*(1) = 2.69, *p* = 0.007, accounting for 10.9% of unique variance (*r*_partial_ = 0.33). FAA was not associated with the outcome, *b* = −0.68, *se* = 4.60, *t*(1) = −0.15, *p* = 0.88.

#### Frontal Alpha Asymmetry Moderation Effect

The multiple regression model with the main effects and interaction effect was significant, *F*_(3_,_63)_ = 4.76, *p* = 0.005, accounting for 18.5% of variance in Externalizing Problems (*R* = 0.43, *R*^2^ = 0.19). Adding the interaction effect resulted in a significant increase in variance accounted for, *R^2^ change* = 0.07, *F change*_(1_,_63)_ = 5.72, *p* = 0.02. Effects in this model are shown in Table 2. The main effect of PDH Total Frequency retained significance (*p* = 0.03), accounting for 8.0% of unique variance. FAA remained unrelated, *p* = 0.78. As hypothesized, there was a significant interaction between PDH Total Frequency and FAA (*p* = 0.02), explaining 8.3% of unique variance in Externalizing Problems.

**TABLE 2:** Parenting daily hassles and frustration task EEG as predictors of externalizing problems.

Terms	Unstandardized beta weight	Std. Error	*t*	*p*	Zero-order *r*	Partial *r*
Intercept	9.05	0.87	10.42	< 0.001		
Frustration task FAA	1.34	4.70	0.29	0.776	0.03	0.04
PDH	0.24	0.11	2.21	0.027	0.33	0.28
FAAxPDH	1.57	0.68	2.30	0.021	0.32	0.29

**FIGURE 1:**
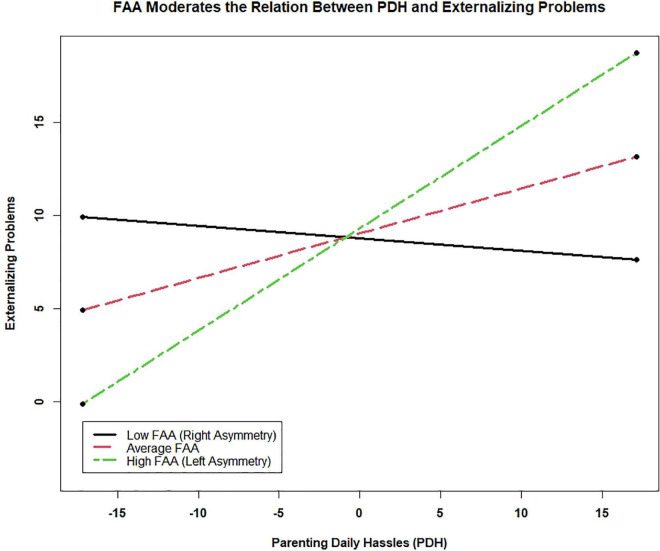
The linear relationship between parenting daily hassles and child externalizing problems is displayed at three levels of the moderator: (1) FAA 1 SD below the mean (i.e., right asymmetry), (2) FAA at the mean, and (3) FAA 1 SD above the mean (i.e., left asymmetry). Parenting daily hassles and FAA were mean centered. The model used for this figure did not contain covariates.

To explore the moderation effect, two additional simple slope regressions were run with FAA linearly transformed to 1 SD above the mean and 1 SD below the mean. When FAA was 1 SD above the mean (i.e., left frontal asymmetry), PDH Total Frequency was a significant predictor, *b* = 0.55, *se* = 0.15, *t*(1) = 3.63, *p* < 0.001, explaining 18.0% of the variance in Externalizing Problems (*r*_partial_ = 0.42); however, when FAA was 1 SD below the mean (i.e., right frontal asymmetry), PDH Total Frequency was not related to Externalizing Problems, *b* = −0.07, *se* = 0.19, *t*(1) = −0.35, *p* = 0.72 (see Figure 1). To determine whether the interaction was more consistent with differential susceptibility or diathesis-stress, the proportion of interaction (PoI) index and proportion affected (PA) index were calculated (Roisman et al., 2012). Consistent with recommendations for PoI, we probed ± 2 SD on the X variable. A PoI index within the range 0.4–0.6 strongly suggests differential susceptibility. The PoI was 0.55. PA indices close to 0.5 strongly indicate differential susceptibility. The PA index was 0.46.

The analysis was rerun while controlling for possible confounding factors. The covariates’ correlations with Externalizing Problems were as follows: child sex [0 = male, 1 = female] (*r* = −0.16, *p* = 0.20), gestational age at birth (*r* = −0.39, *p* = 0.001), maternal anxiety and depression symptoms (*r* = 0.33, *p* = 0.007), maternal age (*r* = −0.13, *p* = 0.30), and gross annual household income [0 = $20k or less, 1 = over $20k] (*r* = −0.14, *p* = 0.38). The model with main effects and covariates was significant, *p* < 0.001, accounting for 38.4% of the variance (*R* = 0.62, *R*^2^ = 0.38). Adding the interaction term resulted in a significant increase in variance accounted for, *R^2^ change* = 0.050, *F change*_(1_,_58)_ = 5.13, *p* = 0.03. PDH Total Frequency accounted for 8.7% of unique variance, *p* = 0.04, *r*_partial_ = 0.30. The PDHxFAA interaction retained significance, *b* = 1.36, *se* = 0.64, *t*(1) = 2.12, *p* = 0.03, *r*_partial_ = 0.28, accounting for 8.0% of unique variance in Externalizing Problems.

### Pokémon-Themed Resting, Eyes-Closed Electroencephalography Condition

#### Main Effects

The main effect model was not significant, *F*_(2_,_65)_ = 2.42, *p* = 0.10.

#### Frontal Alpha Asymmetry Moderation Effect

The multiple regression model with the main effects and interaction effect was not significant, *F*_(3_,_64)_ = 1.85, *p* = 0.15. Coefficients from this model are shown in Table 3. As hypothesized, the interaction between PDH Total Frequency and resting FAA was not significant, *p* = 0.45. When controlling for child sex, gestational age at birth, maternal age, maternal anxiety and depressive symptoms, and gross annual household income, the interaction was still not significant, *b* = −0.70, *se* = 0.75, *t*(1) = −0.94, *p* = 0.35.

**TABLE 3:** Parenting daily hassles and resting EEG as predictors of externalizing problems.

Terms	Unstandardized beta weight	Std. Error	*t*	*p*	Zero-order *r*	Partial *r*
Intercept	8.98	0.80	11.18	< 0.001		
Resting FAA	–2.04	5.83	–0.35	0.727	0.01	–0.05
PDH	0.22	0.10	2.20	0.028	0.26	0.28
FAAxPDH	–0.60	0.79	–0.75	0.453	–0.04	–0.10

### Sensitivity Analyses: Parenting Daily Hassles Parenting Tasks Subscale and Electroencephalography Exclusion

The regression analyses were run again using scores only from the PDH Parenting Tasks Frequency subscale. PDH Parenting Tasks Frequency was correlated with the Externalizing Problems scale (*r* = 0.31, *p* = 0.03). When predicting Externalizing Problems, PDH Parenting Tasks Frequency was a significant predictor (*p* = 0.048, *r*_partial_ = 0.29) but the interaction with frustration task FAA was not, *p* = 0.29. When covariates were added, neither the main effect (*p* = 0.39) nor interaction (*p* = 0.28) were significant. When the resting FAA analysis was repeated, a similar pattern of significance emerged. In the model without covariates, the main effect for PDH Parenting Tasks Frequency was significant (*p* = 0.02) but the interaction between PDH Parenting Tasks Frequency and resting FAA was not, *p* = 0.62. In the model with covariates, neither effect was significant.

One participant used only one mastoid electrode as a reference due to malfunctioning of the other. Because this asymmetry could have biased the FAA calculation, we reran the analysis excluding this participant to see if results changed. When this participant was excluded, we replicated the exact patterns of significance/non-significance for the main and interaction effects in all regressions reported in Sections 3.2.2–3.4.

## Discussion

The aim of this study was to examine the relationship between daily parenting hassles, child FAA, and child externalizing problems in a sample of predominantly African American mother-child dyads living in an under-resourced urban ecology. The study was partially guided by the PPCT model of Bronfenbrenner’s Bioecological Theory, leading to hypotheses about the interaction of proximal processes, person characteristics, and contextual factors. Results revealed that the mothers’ frequency of daily parenting hassles were positively correlated with their child’s externalizing behaviors. As hypothesized, child FAA moderated this relationship, but only when measured during a frustration task. Among children with more left asymmetry, their mother’s daily parenting hassles were associated with their externalizing problems, but this association was not found among children with more right asymmetry. This effect was consistent with differential susceptibility rather than diathesis-stress.

These results align with previous studies establishing FAA as a moderator of emotional processes and support the motivational direction hypothesis, mapping left/right asymmetry on to approach/withdrawal motivation (Rutherford and Lindell, 2011; Harmon-Jones and Gable, 2018; Reznik and Allen, 2018). They also extend the comparatively small portion of the literature linking approach motivation to maladaptive behavior patterns (Minnix et al., 2004; Peterson et al., 2008; Hale et al., 2009; Keune et al., 2012, 2015; Beeney et al., 2014; Nusslock et al., 2015; Ellis et al., 2017). Because FAA functioned as a moderator when measured during a frustration task but not during resting state, results lend support to the capability model, which argues that FAA is more validly and usefully construed as an index of a capability marshaled in response to the demands of a specific situation rather than a tendency to respond the same way in most or all situations. To our knowledge, this is the first study establishing an interaction between child FAA and parental perception of daily parenting hassles. This raises important questions as to how FAA interacts with family stress and emotional processes to produce problem behaviors, especially given that FAA is associated with a variety of psychosocial risk factors but does not straightforwardly predict internalizing or externalizing problems in children (Peltola et al., 2014).

### Parenting Daily Hassles and Child Externalizing Problems

Our study replicated the finding of previous studies that mothers who report more frequent daily parenting hassles tend to also report more externalizing problems for their children (Kliewer and Kung, 1998; Coplan et al., 2003; Crnic et al., 2005; Yaman et al., 2010; Stone et al., 2016; Walerius et al., 2016). In our sample, hassles explained 8% of unique variance in externalizing problems. Importantly, this is a correlation that gives no information on causation or the direction of the effect. This association between parental stress and child adjustment problems has been explained with the Family Stress Model, wherein parental stress indirectly stresses the child *via* disrupted parenting behaviors, leading to behavioral problems. Creasey and Reese (1996) found the relationship held after controlling for non-parenting daily hassles, suggesting a specific link between child behavior problems and daily parenting hassles rather than simply total stress experienced by the parent. One possibility is that PDH Total Frequency and CBCL Externalizing Problems scales may be measuring the same construct.

The PDH Total Frequency scale is comprised of the Parenting Tasks Frequency and Challenging Behavior Frequency subscales. Because the Challenging Behavior Frequency subscale asks parents to report on parenting hassles directly related to challenging child behavior, it could be argued that it verges on conceptual redundancy with parent reports of child externalizing problems. In addition to shared methods variance, both scales have items measuring how often children won’t listen and resist their bedtime. The Parenting Tasks Frequency subscale, on the other hand, asks parents to report how often they engage in potentially stressful parenting tasks that are not (with some exceptions) in direct response to challenging child behaviors. The items are more centered on their experience as a parent, sometimes measuring the extent to which they feel their parenting tasks detract from the rest of their life (e.g., child interfering with other household needs, having to change your plans because of child, etc.). We found that the Parenting Tasks Frequency subscale was moderately correlated with externalizing problems, as was the total frequency scale. In the regressions with covariates, the PDH Total Frequency scale was significantly associated with child externalizing problems, but the PDH Parenting Tasks Frequency subscale was not. Taken together, these results suggest that none of the PDH scales are conceptually redundant with CBCL Externalizing Problems, and mothers’ subjective experience of the frequency of hassling parenting tasks does predict externalizing problems, though not as strongly as when the more conceptually similar challenging behaviors scale is included. Crucially, the PDH scales measure parents’ subjective perception. While this introduces error, Bronfenbrenner pointed out the importance of conceptualizing the phenomenological field as part of the ecology, making the subjective impression and indeed the “error” meaningful and perhaps explanatory. For example, mothers with higher cognitive appraisal errors (both negative cognitive errors and positive illusions) reported much more frequent daily parenting hassles (Mazur, 2006), indicating that the “error” with respect to objective counting is partially a valid measure of an attributional disposition linked to mental health outcomes.

Given abundant research showing a reciprocal, bidirectional relationship between parent and child functioning, the association we found between parenting hassles and child externalizing problems was also likely reciprocal and bidirectional. Previous research has found externalizing-spectrum behaviors are stressful for parents, especially during early childhood, and the direction from child to parent has sometimes been shown to be stronger than the converse (Lansford et al., 2011; Stone et al., 2016; Walerius et al., 2016). Previous studies have found that the relationship between parenting hassles and child problem behaviors is mediated by warmth and harsh discipline (Gülseven et al., 2018). The Bioecological Theory suggests that these mediators likely depend on dispositions of the parents, the demand characteristics of the child, a variety of contextual factors, and their interactions. For example, more daily hassles have predicted less sensitive parenting only for parents with genetic predispositions toward inefficient dopamine processing (van IJzendoorn et al., 2008). While some studies report elevated authoritarian parenting in Black/African American mothers, especially those in low-SES contexts, other studies have found this parenting to be adaptive in Black/African American samples (whereas it is maladaptive for White European Americans), likely because it is intended to protect the child from dangers of racism, especially those they might face when experiencing externalizing problems in front of authority figures like teachers and police (Pearl et al., 2012; Dunbar et al., 2021). Thus, additional research is needed to understand mediators, moderators, and complex dynamics that are likely to causally connect parenting hassles to child externalizing problems and later psychosocial adjustment, both generally and in African American families living in under-resourced urban areas.

### Frustration Task Frontal Alpha Asymmetry as a Moderator

As we hypothesized, FAA moderated the relationship between daily parenting hassles and externalizing problems. While the overall effect size was small (8% of unique variance), the difference between levels of the moderator was large. Frequency of parenting daily hassles accounted for 18% of the variance in externalizing problems for dyads with children producing FAA 1 SD above the mean (i.e., left asymmetry) during the frustration task but was not related to externalizing problems when FAA was 1 SD below the mean (i.e., right asymmetry). FAA did not directly predict externalizing problems. We interpret these findings as confirmation of the motivational direction hypothesis and the role of FAA as a shaper and potentiator of risk factors for externalizing problems rather than a risk factor on its own.

Frontal alpha asymmetry was measured during a task designed to elicit developmentally appropriate frustration in children *via* the presentation of uncontrollable and unpredictable negative feedback during a computer game (Grabell et al., 2019). FAA was measured during 2-s windows directly following the presentation of audiovisual negative feedback stimuli. We interpret left asymmetry during these windows as an unconscious approach-oriented motivational response to a social-evaluative stressor and right asymmetry as a withdrawal response. Because the task was designed to elicit frustration and frustration is an approach-oriented emotion, one plausible interpretation is that left asymmetry in this paradigm marked both a tendency to respond to environmental stressors with the approach motivational system and more specifically, anger, which previous studies have found (Harmon-Jones and Allen, 1998; Harmon-Jones, 2007). Our interpretation also aligns well with prior studies linking left asymmetry to aggression (Peterson et al., 2008; Keune et al., 2012). Further, it was possible to look at continuous ratings of mood given by children to explore whether the task was associated with a downward mood trend and whether mood was lower after negative feedback than after positive feedback. We replicated the findings of Grabell et al. (2019) that average self-rated mood during the task was slightly positive and the expected decrease in mood during the task did not occur; however, when we took the rating after the practice trials into account, we did see a decrease. In our sample, average emotional valence was positive immediately prior to the game, dropped to a slightly positive level after trial 10, then remained at about that same level after trials 20 and 30. Overall, these ratings provided evidence of a frustration effect because mood decreased during the task relative to a baseline rating obtained after the practice round and average emotional valence was lower after negative feedback than positive feedback. Moreover, Grabell et al. (2019) coded facial expressions after negative feedback and found evidence of induced negative affect. Although we did not code facial expressions, a powerful approach for future studies will be to use multiple additional measures of affect during the Incredible Cake Kids task, such as facial expressions and psychophysiological responses, as these are not subject to reporting bias and, in the case of children, less subject to immature articulation of emotional states.

Frontal alpha asymmetry during the frustration task was not directly correlated with externalizing problems but was related to externalizing problems in interaction with daily parenting hassles. Results from two previous studies of child externalizing problems share this same generic structure, one where a dispositional variable relevant to externalizing problems (in this case inattentive temperament) interacted with parenting stress to predict externalizing problems in preschoolers (Coplan et al., 2003) and another where a dispositional variable (in this case FAA) interacted with physiological reactivity to predict externalizing problems in African American children with low SES (Gatzke-Kopp et al., 2014). Our results strongly support the interaction as an instance of differential susceptibility (Belsky and Pluess, 2009) rather than diathesis stress. This means that left asymmetry ranged from adaptive to maladaptive depending upon the level of parenting daily hassles. Children with left asymmetry had significantly more externalizing problems than those with right asymmetry when parenting daily hassles were high; however, when parenting daily hassles were low, those with left asymmetry had significantly *fewer* externalizing problems than those with right asymmetry. Because parenting hassles likely detract from parents’ ability to structure their child’s environment with adaptive operant conditioning, it is plausible that left asymmetry is adaptive in low hassle family environments with a dearth of maladaptive positive distractors. On the other hand, left asymmetry may be maladaptive in high hassle family environments where positive distractors are more prevalent and detract from appropriate goal-directed behavior for children with higher approach motivation. This is in contrast to children with right asymmetry, for whom parenting hassles were not associated with externalizing problems. For these children, their inability to disengage attention from maladaptive negative information (i.e., the audio-visual negative feedback) may have activated a withdrawal response, thus protecting them from externalizing problems.

In conjunction with similar studies showing FAA functioning as a moderator, our study helps explain why meta-analyses have not found support for FAA as a risk factor for externalizing problems in children (Peltola et al., 2014; Perizzolo et al., 2017). We lack the requisite causal evidence to determine if left asymmetry exacerbated the link between parental stress and externalizing problems or if right asymmetry attenuated it. Given that previous studies have reported positive associations between parenting hassles and externalizing problems, it may be more likely that right asymmetry attenuated this link. While right asymmetry might have protected against externalizing problems, this should not be confused with a generalized protective effect. For example, while we did not test this hypothesis, right asymmetry could have exacerbated the link to internalizing problems. A straightforward interpretation of our moderation effect from a Family Stress Model allowing for bidirectional influence would be that stress—whether in the parent, child, or both—interacts with the child’s motivational disposition to shape a pattern of dysfunctional behavioral responding; however, the PPCT model introduces contextual considerations that complicate this interpretation. It may be the case that this stress by left asymmetry interaction only leads to externalizing problems (or any type of dysfunction) in dyads with mothers subjected to precarious employment or workplace exploitation (i.e., exosystem factors) or families living in urban poverty and/or facing minority stress (i.e., macrosystem factors). However, our sample either lacked data on salient factors or was too homogenous to compare dyads across these circumstances.

Results also showed differential functions for FAA depending on the measurement condition. While neither frustration task FAA nor resting FAA were related to externalizing problems, FAA measured during the frustration task functioned as a moderator while resting FAA did not. At minimum, this highlights the strength of the Incredible Cake Kids frustration task in eliciting asymmetric PFC activity (indexed by F3/F4 electrodes) which, in interaction with parenting hassles, has superior predictive validity for child externalizing problems compared to resting FAA measured in the Pokémon-themed resting condition. This suggests that future studies with similar aims may benefit from utilizing the Incredible Cake Kids task or creating a similar one, perhaps designed to elicit a variety of affects within and/or between motivational directions so that they can be comparatively studied. It also suggests that task-elicited FAA may interact with different forms of stress outside of parenting hassles to predict problem behaviors or interact with similar environmental risk factors such as household chaos, parental mental health conditions, or neighborhood characteristics.

In our study, we found that task-elicited and resting FAA were only moderately correlated. This strongly suggests that the two tasks were measuring different processes. Though we did not measure child affect during resting FAA, differences in evoked affect may have partially accounted for differences in FAA values across conditions. To our knowledge, Goodman et al. (2013) and our study belong to a small minority of FAA studies that report an association between resting and task-elicited FAA. We can find no reviews or meta-analyses on the relation between these measures, the construct(s) they putatively assess, or their discriminant and convergent validity. Thus, the relationships between resting state asymmetry and many hypothetical constructs involving asymmetric frontal activity/activation in response to emotional challenges are unknown. These distinctions are important because if resting state FAA does operationalize a different construct than various types of emotional challenge activation, one would expect some non-zero portion of the non-significant associations with resting state asymmetry to obtain significance when associated with asymmetry during ecologically valid emotional challenges. Moreover, because the capability model leaves open the definition of an “emotional challenge” by not specifying standardized stimuli/tasks and measurement conditions, it leaves open which capabilities (i.e., lateralized neuropsychological functions involving the dlPFC) will be demanded. Thus, a collection of studies following the capability model are not in virtue of that fact studying the same or even closely related neuropsychological construct(s).

### Limitations and Future Directions

Our study has important limitations that bear mentioning. First, all measures were collected at the lab visit when the child was approximately 5 years old, making prediction concurrent rather than prospective. Therefore, no causal claims can be made. Second, while we cited the PPCT model as a guiding force, our study completely left out issues of micro-, meso-, and macro-time. Though this study was drawn from a larger multi-year longitudinal study and is amenable to deeper investigations of development using the PPCT model, EEG was only measured at the 5-year visit. Our sample was also relatively small and very homogenous, limiting power and eliminating the possibility of studying the effects of variability in pertinent contextual and demographic factors. While we examined an understudied and underserved sample in a high-risk low-SES urban context, due to the relative homogeneity of the sample we did not study how variation in neighborhood factors, income, or wealth may have modified our results. We studied child behavior only as observed by the mother and thus, primarily only in the microsystem of the home. We do not know whether or how the observed interaction between FAA and parenting hassles may have related to behavior in the classroom or on the playground. Parent daily hassles and externalizing problems were both operationalized through maternal self-report, leaving open the possibility that some significant portion of their association was explained by shared methods variance and reporting bias.

There were limitations to our EEG methods and FAA analysis. The Incredible Cake Kids EEG task was designed to induce frustration in response to unavoidable negative feedback (Grabell et al., 2019). Although our child self-report data provided some evidence of this frustration effect, prior evidence shows young children’s self-reports of their emotional states are unreliable and correlate weakly with a “gold-standard” measure of emotion (i.e., rating of facial expressions by trained coders) (Zeman et al., 2007; Durbin, 2010). This methodological limitation casts some doubt on our interpretations of the meaning of FAA in response to negative feedback and its interaction with parenting daily hassles. Future studies should check the intended frustration effect by measuring facial expressions and/or psychophysiological responses. Furthermore, we used an average of 29.8 s of artifact-free EEG data to calculate FAA in response to the negative feedback trials, which is well below an expert recommendation of one to 3 min needed for excellent reliability (Smith et al., 2017). This increases the probability that our FAA measure in response to negative feedback was biased by measurement error. The shortage of artifact-free epochs resulted from the design of the Incredible Cake Kids task, which presents the negative audiovisual feedback for a total of 36 s. Future studies should modify the task to include additional trials or use other tasks offering a bare minimum of 2 min of target data in anticipation of possible substantial data loss due to artifact rejection. Next, the Pokémon-themed EEG condition was designed to facilitate affectively neutral, resting behavior and elicit resting-state brain networks; however, it may have activated reward anticipation neurocircuitry and corresponding lateralized cortical-frontal activity. Children may have found the egg hatching animation, sound effect, and Pokémon sticker to be rewarding, especially if they were fans of Pokémon, excited by stickers, and/or sensitive to positive social feedback. We did not measure children’s affective response to this task or the extent to which they perceived the consequents of their quiescent behavior to be rewarding. FAA has been studied as a marker of reward sensitivity, with increased relative left frontal activity associated with increased reward anticipation and curiosity, two forms of approach motivation (Gorka et al., 2015; Käckenmester et al., 2018). Thus, our putative resting-state FAA measure may better be interpreted as a measure of reward anticipation FAA, though more data is needed. If this were the case, we would still not expect FAA during the Pokémon-themed task to function as a moderator of parenting hassles and externalizing problems, though it may relate to internalizing problems, given the link between depression and reduced reward sensitivity.

A substantial proportion of our participants were missing EEG data for a variety of reasons. This may have narrowed our sample in a systematic fashion, limiting generalizability to the predominately low-SES urban African American population from which our sample was drawn. Given that psychological research lacks diversity (Nielsen et al., 2017; Roberts et al., 2020), and prior research has linked SES to differential patterns of brain development such as EEG power (Brito et al., 2016), these questions need further investigation within this historically understudied population; however, the current study is limited in the generalizability of its findings for this same reason. In addition, racial and ethnic heterogeneity and genetic variability were not controlled. Variation in socially constructed cultural and/or genetic factors may have modulated the independent and dependent variables and may limit generalizability. We also limited our EEG power spectral analysis to the alpha band, leaving other asymmetries and other potentially pertinent factors such as delta-beta coupling unexamined. Next, FAA is a measure of relative hemispheric differences in alpha power that does not account for effects of absolute power levels or which hemisphere drove the asymmetry score (i.e., left asymmetry resulting from below average right activity or above average left activity). It may be the case, for example, that absolute levels of alpha power at one or both frontal electrodes were related to hassles and/or externalizing problems and this variance was not accounted for. Finally, the mean FAA value in our sample was slightly asymmetric to the left. Because we analyzed FAA variability around the sample mean rather than classifying individuals into left/right asymmetry groups, our results may not necessarily hold for samples with asymmetry distributions centered around zero or right asymmetry. While most FAA studies analyze the data as we did, this may still limit generalizability. More broadly, because EEG has low spatial resolution and the dlPFC is implicated in a variety of cortico-limbic cognitive-affective processes, our analysis provided no insight into the complex interactions among brain networks responsible for emotional processing.

Future studies can learn from the strengths and limitations of our study. While our study found support for FAA as a moderator when measuring parent stress with self-report, it would be interesting to see if the same result was obtained with analysis of cortisol levels. The role of FAA as a moderator between stress and maladaptive developmental outcomes should be further investigated with a focus on teasing apart the roles of both left and right asymmetry. A modified version of the Incredible Cake Kids frustration task allowing for at least 2 min of negative feedback EEG data may be worth developing for future studies of task-elicited FAA, especially those investigating anger processes. More generally, future investigators interested in FAA can benefit from viewing it as a *type* of measurement that can measure a wide variety of neuropsychological capabilities rooted in dlPFC activity depending on the unique, replicable environmental contingencies constituting the “emotional challenge” during which the EEG is recorded. Future studies can also utilize the Bioecological Theory and PPCT model to expand upon present findings. For example, relatively little is known about what factors drive the development of FAA. Multiple studies have shown low heritability estimates (Coan, 2003; Anokhin et al., 2006), making investigation into environmental effects promising. Longitudinal studies measuring FAA in multiple conditions at multiple time points could answer developmental questions using structural equation modeling and path analyses. Finally, the FAA literature could benefit from a meta-analysis and/or systematic review contrasting the associations of FAA measured in various conditions and situating the neuropsychological constructs FAA putatively operationalizes within the nomological network (Cronbach and Meehl, 1955).

## Conclusion

This study found that FAA does not individually predict externalizing problems in children but does so in interaction with parental daily hassles. It demonstrated the incremental predictive validity of FAA measured in a relatively novel frustration task, as the interaction effect was not significant for resting state FAA. This finding in conjunction with the small correlation between the two measures of FAA raise important psychometric questions. The moderation effect for frustration task FAA showed that while there was a medium-sized positive correlation between parenting hassles and child externalizing problems for children with left asymmetry, there was no relationship between these factors for children with right asymmetry. More generally, these results shed light on conditions in which approach motivation can function adaptively or maladaptively relative to withdrawal motivation.

## Data Availability Statement

The raw data supporting the conclusions of this article will be made available by the authors, without undue reservation.

## Ethics Statement

The studies involving human participants were reviewed and approved by Wayne State University Institutional Review Board. Written informed consent to participate in this study was provided by the participants or their legal guardian/next of kin.

## Author Contributions

DM, MT, CT, and MH contributed to the conception and design of the study. MH and DM processed the EEG data and AP helped code EEG videos. DM performed the statistical analyses and wrote the abstract, introduction, results, and discussion sections of the manuscript. AP wrote the materials and methods section and contributed to the discussion section. All authors contributed to the manuscript review and revision and approved the submitted version.

## Conflict of Interest

The authors declare that the research was conducted in the absence of any commercial or financial relationships that could be construed as a potential conflict of interest.

## Publisher’s Note

All claims expressed in this article are solely those of the authors and do not necessarily represent those of their affiliated organizations, or those of the publisher, the editors and the reviewers. Any product that may be evaluated in this article, or claim that may be made by its manufacturer, is not guaranteed or endorsed by the publisher.
